# Association of DPP4 Gene Polymorphisms with Type 2 Diabetes Mellitus in Malaysian Subjects

**DOI:** 10.1371/journal.pone.0154369

**Published:** 2016-04-25

**Authors:** Radwan H. Ahmed, Hasniza Zaman Huri, Zaid Al-Hamodi, Sameer D. Salem, Boshra Al-absi, Sekaran Muniandy

**Affiliations:** 1 Department of Molecular Medicine, Faculty of Medicine, University of Malaya, Kuala Lumpur, Malaysia; 2 Department of Pharmacy, Faculty of Medicine, University of Malaya, Kuala Lumpur, Malaysia; 3 Clinical Investigation Centre, University Malaya Medical Centre, Kuala Lumpur, Malaysia; 4 Department of Biochemistry and Molecular Biology, Faculty of Medicine, Sana’a University, Sana’a, Yemen; University of Leuven, Rega Institute, BELGIUM

## Abstract

**Background:**

Genetic polymorphisms of the Dipeptidyl Peptidase 4 (DPP4) gene may play a role in the etiology of type 2 diabetes mellitus (T2DM). This study aimed to investigate the possible association of single nucleotide polymorphisms (SNPs) of the DPP4 gene in Malaysian subjects with T2DM and evaluated whether they had an effect on the serum levels of soluble dipeptidyl peptidase 4 (sDPP-IV).

**Method:**

Ten DPP4 SNPs were genotyped by TaqMan genotyping assays in 314 subjects with T2DM and 235 controls. Of these, 71 metabolic syndrome (MetS) subjects were excluded from subsequent analysis. The odds ratios (ORs) and their 95% confidence interval (CIs) were calculated using multiple logistic regression for the association between the SNPs of DPP4 and T2DM. In addition, the serum levels of sDPP-IV were investigated to evaluate the association of the SNPs of DPP4 with the sDPP-IV levels.

**Results:**

Dominant, recessive, and additive genetic models were employed to test the association of DPP4 polymorphisms with T2DM, after adjusting for age, race, gender and BMI. The rs12617656 was associated with T2DM in Malaysian subjects in the recessive genetic model (OR = 1.98, *p* = 0.006), dominant model (OR = 1.95, *p* = 0.008), and additive model (OR = 1.63, *p* = 0.001). This association was more pronounced among Malaysian Indians, recessive (OR = 3.21, *p* = 0.019), dominant OR = 3.72, *p* = 0.003) and additive model (OR = 2.29, *p* = 0.0009). The additive genetic model showed that DPP4 rs4664443 and rs7633162 polymorphisms were associated with T2DM (OR = 1.53, p = 0.039), and (OR = 1.42, p = 0.020), respectively. In addition, the rs4664443 G>A polymorphism was associated with increased sDPP-IV levels (*p* = 0.042) in T2DM subjects.

**Conclusions:**

DPP4 polymorphisms were associated with T2DM in Malaysian subjects, and linked to variations in sDPP-IV levels. In addition, these associations were more pronounced among Malaysian Indian subjects.

## Introduction

Type 2 diabetes mellitus (T2DM), which is a complex metabolic disease that results from peripheral insulin resistance and reduced insulin secretion [1], comprises about 90% of the global cases of diabetes mellitus (DM) [2]. According to the International Diabetes Federation (IDF), nearly 387 million people worldwide were affected by diabetes by 2014, and this number is projected to rise to 592 million people by 2035 (10% of the adult population) [3]. In Malaysia, there were 3.2 million cases of diabetes by 2014 (16.6% of the adult population) [3].

Genetic factors and lifestyle play a critical role in the development of T2DM [4].The Asian population has strong genetic susceptibility to T2DM, developing diabetes at younger ages and at a lower degree of obesity [5]. Currently the incidence of T2DM has reached epidemic levels in Asia [6]. Despite knowledge of the critical role of genetic factors, these have not been incorporated into the clinical assessment of T2DM risk [4].

The DPP4 gene encoding for dipeptidyl peptidase IV (DPP-IV), also known as cell surface antigen CD26, is located on chromosome 2q24.3 [7]. DPP-IV is a type II transmembrane protein that is cleaved off and released into the circulation as a soluble form (sDPP-IV) through the shedding process [8–10], to be implicated in the catalytic degradation of incretins, such as glucagon-like peptide-1 (GLP-1), which play an essential role in the glucose homeostasis [11–13]. Accordingly, the DPP-IV has recently gained medical importance since DPP-IV inhibitors have been approved for the treatment of T2DM [14]. It is noteworthy that DPP-IV is a predictor for insulin resistance and MetS in normal subjects [15], and has a key role in the pathophysiology of T2DM by regulating incretins [16]. It also has a nonenzymatic function as a binding and signaling protein, suggesting a teleological role in inflammation and cardiovascular regulation [17].

Our recent work suggests that sDPP-IV levels were increased in Malaysian patients with T2DM and correlated negatively with the levels of active GLP-1 [18]. In this respect, increased expression, serum levels and activity of DPP-IV on T-cells have also been reported in T2DM [19].

In the visceral adipose tissue (VAT), the DPP4 tagging SNPs was associated with methylation of DPP4 and affected mRNA abundance, and, in severe obesity, was a risk factor for cardiovascular disease [20, 21]. In addition, polymorphism in the DPP4 gene was a risk factor for a myocardial infarction (MI) in patients of European ancestry with atherosclerosis [22]. Nonetheless, genetic polymorphisms of DPP4 and their association with T2DM have rarely been investigated [14]. Moreover, a review of the scientific literature revealed that the association of the DPP4 genetic polymorphisms gene with T2DM among Asian populations has not been published. To the best of our knowledge, no such study has been initiated in Asian populations.

Hence, this study was performed to investigate whether DPP4 gene polymorphisms are associated with T2DM in Malaysian subjects and to determine SNPs within the DPP4 gene, which could be associated with serum sDPP-IV levels.

## Materials and Methods

### Ethics Statement

This study complied with the principles set out in the ethical guidelines of the Declaration of Helsinki. Additionally, all the patients and control subjects signed written informed consent before commencement of the study, which was designed, and received the approval of the Medical Ethics Committee of the University Malaya Medical Centre (UMMC), (approval number 387.15).

### Subjects, demographic and biochemical analysis

This hospital-based case-control study involved T2DM patients, who were defined according to the World Health Organization diagnostic criteria [23, 24] by the attending physicians/endocrinologists and who were in the follow-up treatment at the UMMC, Kuala Lumpur. The control subjects enrolled were selected from those who attended the UMMC for a routine medical check-up, and who had a fasting plasma glucose of equal to or less than 100 mg/dL (6.1 mmol/L). Pathological and normal were included with each run of biochemical analysis. Any patients receiving DPP-IV inhibitors, or who had acute or chronic diseases of the heart liver or kidney, or who had a malignancy were excluded from this study. Details regarding the data collection, anthropometric measurements, demographic parameters, and biochemical analysis have been reported previously [18].

### Measurements of serum sDPP-IV concentration

The level of sDPP-IV in the fasting serum from each participant was quantified by the enzyme-linked immunosorbent assay method using the Human CD26 (RayBiotech, Inc., USA) according to the manufacturer’s protocol. Colorimetric quantification at 450 nm was performed with a microplate reader (Hydroflex Elisa, Chemopharm, Austria).

### Genomic DNA extraction and SNP genotyping

Genomic DNA from each subject was extracted from the peripheral blood leukocytes using a commercially Wizard^®^ Genomic DNA Purification Kit (Promega Corporation, Madison, WI, USA) according to the instructions of the manufacturer. We selected seven SNPs in the DPP4 gene (rs3788979, rs1558957, rs1861978, rs4664443, rs12617656, rs17574, rs7608798) based on previous indications that were studied with risk factors for T2DM, and one SNP (rs7633162) was associated with DPP-IV inhibition. In addition, we selected two SNPs (rs2160927, rs1014444) with a minor allele frequency of > 0.10, for a more complete coverage of the DPP4 gene. Genotyping of all 10 SNPs was performed with pre-designed TaqMan^®^ genotyping assay 40x (available from Applied Biosystems of Life Technologies, Foster city, CA, USA). All of the assays were conducted in 96-well PCR plates using the StepOnePlus Real-Time PCR system (Applied Biosystems Inc, USA) according to the manufacturer’s instructions. The TaqMan call rates for genotyping was over >99% and DNA samples were genotyped in duplicate with 100% concordance, and included negative controls in each run to verify and ensure the accuracy of the genotyping results.

### Statistical Analysis

Demographic and biochemical characteristics were presented as means ± standard deviation (SD). Hardy-Weinberg equilibrium (HWE) analyses were applied to compare the observed and expected genotype frequencies among diabetes-free controls by a goodness-of-fit chi-square using the DeFinetti software (http://ihg.gsf.de/cgi-bin/hw/hwa1.pl) from the Institute of Human Genetics. The Golden Helix SNP & Variation Suite (SVS v8.x software; Bozeman, MT, USA) was used to estimate the linkage disequilibrium (LD) between SNPs and calculate the D′ and r^2^.

All the statistical tests were performed using SPSS v. 20 (IBM SPSS Inc., Chicago, IL, USA). Odds ratio (ORs), 95% (CI) and the corresponding *P* values were calculated using multiple logistic regression analysis to assess the association of each SNP, with different genetic models (dominant, recessive, and additive) in all T2DM subjects and also with T2DM in each ethnic group. Multiple testing was performed using the Benjamini-Hochberg false discovery rate (FDR) [25], and the significance level was set at 5%. The association of genotype groups in DPP4 (SNPs) with sDPP-IV levels were performed using the general linear model with a Bonferroni adjustment. P-values < 0.05 were considered statistically significant.

## Results

### Demographic and biochemical parameters

The study included 314 subjects with T2DM and 235 non-diabetic (control) subjects. After we performed biochemical tests on all subjects and applied (MetS) criteria [26] on the non-diabetic control participants it revealed that 71 subjects had MetS. Metabolic syndrome is a risk factor for T2DM that could have an impact on association studies and (HWE). Consequently, 71 subjects in the control group with MetS were excluded from further analysis. Hence, 164 subjects were recruited as control without either diabetes mellitus or MetS.

The demographics and the biochemical parameters of all subjects are shown in Table 1. A significant difference was found between the case and the control groups in both the diabetic and metabolic parameters (Table 1).

**Table 1:** Demographic and biochemical parameters of type 2 diabetes mellitus and control subjects.

Parameters	Control (n = 164)	Type 2 diabetes (n = 314)	P-Value
Gender % (Male/Female)	39.0/61.0	46.5/53.5	
Races % (Malay/Chinese/Indian)	52.4/31.7/15.9	48.7/16.9/34.4	
Age (years)	50.03 ± 12.30	51.25 ± 7.86	*0*.*191*
Weight (kg)	61.94 ± 13.9	71.94 ± 15.5	***<0*.*001***
Height (m)	1.62 ± 0.1	1.59 ± 0.1	***0*.*004***
Body Mass Index (kg/m^2^)	23.68 ± 4.2	28.51 ± 5.4	***<0*.*001***
Waist Circumference (cm)	82.41 ± 12.4	95.38 ± 12.2	***<0*.*001***
Systolic Blood Pressure (mmHg)	131.5 ± 19.7	135.8 ± 19.4	***0*.*026***
Diastolic Blood Pressure (mmHg)	81.1 ± 9.9	82.3 ± 10.4	*0*.*167*
Fasting Plasma Glucose (mmol/L)	5.02 ± 0.4	8.46 ± 3.7	***<0*.*001***
Glycosylated A1c (%)	5.59 ±0.4	8.37 ±2.2	***<0*.*001***
Fasting Plasma Insulin (pmol/L)	57.53 ± 37	128.9 ± 86.4	***<0*.*001***
Homeostasis model assessment-insulin resistance HOMA-IR	1.23 ± 0.8	3.50 ± 3.1	***<0*.*001***
Total-Cholesterol (mmol/L)	5.26 ±1.0	4.85 ± 1.1	***<0*.*001***
High Density Lipoprotein (mmol/L)	1.54 ± 0.4	1.18 ± 0.3	***<0*.*001***
Triglyceride (mmol/l)	1.15 ± 0.6	1.88 ± 0.6	***<0*.*001***

The results presented represent as mean ± standard deviation. Bold values are significant.

### Association between DPP4 polymorphisms and T2DM

The observed genotype frequencies for all DPP4 SNPs were consistent with (HWE) P-value >0.05 in the control subjects, except for one SNP rs3788979, *p* = 0.031. Hence, rs3788979 was excluded from the subsequent analysis.

Dominant, recessive, and additive genetic models were employed to test the association of DPP4 polymorphisms with T2DM using the multiple logistic regression analysis after adjusting for age, race, gender and BMI. The SNP rs12617656 was associated with T2DM, in Malaysian subjects under the recessive genetic model (OR (95%CI) = 1.98(1.22–3.20); *p* = 0.006), dominant model OR (95%CI) = 1.95(1.19–3.19); *p* = 0.008), and additive model OR (95%CI) = 1.63(1.22–2.19); *p* = 0.001). For the SNP rs4664443, it exhibited a significant association with T2DM, which was observed in the additive model OR (95%CI) = 1.53 (1.02–2.28); *p* = 0.039). Likewise, the SNP rs7633162 was associated with T2DM in the additive model OR (95%CI) = 1.42(1.06–1.90); *p* = 0.020). However, the associations for rs4664443 and rs7633162 did not remain significant after controlling for a false discovery rate of 5%. In addition, rs7633162 showed a border line association under the recessive OR (95%CI) = 1.56(1.00–2.46); *p* = 0.053), and dominant model OR (95%CI) = 1.67(1.00–2.82); *p* = 0.052).

However, no association was found between T2DM and other SNPs rs1558957, rs1861978, rs2160927, rs17574, rs7608798, rs1014444 (Table 2).

**Table 2:** Association of DPP4 polymorphisms with type 2 diabetes mellitus among Malaysian subjects.

	Control		Type 2 diabetes		Recessive			Dominant			Additive		
DPP4 SNPs	Freq.	11/12/22	Freq.	11/12/22	OR (95% CI)	P-Value	P-Value*	OR (95% CI)	P-Value	P-Value*	OR (95% CI)	P-Value	P-Value*
rs7608798 (A < **G**)	0.54	39/75/50	0.61	58/133/123	1.32 (0.84–2.09)	0.235	0.026	1.01 (0.60–1.70)	0.959	0.050	1.13 (0.84–1.45)	0.429	0.039
rs1014444 (G < A)	0.49	46/76/42	0.59	62/137/115	1.40 (0.87–2.26)	0.169	0.022	1.23 (0.75–2.02)	0.410	0.037	1.22 (0.91–1.64)	0.178	0.024
rs12617656 (C < T)	0.46	54/71/39	0.62	52/133/129	1.98 (1.22–3.20)	**0.006**	0.004	1.95 (1.19–3.19)	**0.008**	0.006	1.63 (1.22–2.19)	**0.001**	**0.002**
rs7633162 (G < C)	0.54	42/69/53	0.61	55/132/127	1.56 (1.00–2.46)	0.053	0.013	1.67 (1.00–2.82)	0.052	0.011	1.42 (1.06–1.90)	**0.020**	0.007
rs4664443 (G > A)	0.13	125/34/5	0.21	199/96/19	2.21 (0.75–6.54)	0.153	0.020	1.60 (0.98–2.62)	0.061	0.015	1.53 (1.02–2.28)	**0.039**	0.009
rs2160927 (T < C)	0.52	43/72/49	0.63	57/131/126	1.29 (0.82–2.05)	0.276	0.033	1.21 (0.73–2.01)	0.464	0.041	1.18 (0.88–1.58)	0.268	0.031
rs17574 (T < C)	0.84	5/44/115	0.86	6/75/233	1.34 (0.83–2.16)	0.238	0.028	1.36 (0.34–5.34)	0.663	0.046	1.29 (0.84–1.96)	0.244	0.030
rs1861978 (G < T)	0.89	3/30/131	0.90	4/55/255	1.56 (0.89–2.74)	0.119	0.017	1.61 (2.95–8.82)	0.581	0.044	1.48 (0.90–2.43)	0.123	0.019
rs1558957 (C > T)	0.10	135/26/3	0.33	258/47/9	2.03 (0.50–8.33)	0.325	0.035	0.85 (0.48–1.50)	0.563	0.043	0.98 (0.61–1.55)	0.916	0.048

Risk allele frequency (Freq.) and genotype counts in individuals with type 2 diabetes and control subjects. In the additive model, genotype of homozygote for the non-risk allele (11), heterozygote (12) and homozygote for the risk allele 22. The recessive model was defined as 22 versus 12 + 11 and dominant model as 22+12 versus 11. The results are presented as odds ratio, 95% CI, and P value adjusted for age, race, gender and BMI using logistic regression. The outliers (studentized residual is greater than 2.0 or less than −2.0) were excluded.

* Adjusted for multiple comparisons using the false discovery rate at the 5% level. Bold values are significant.

**Fig 1:**
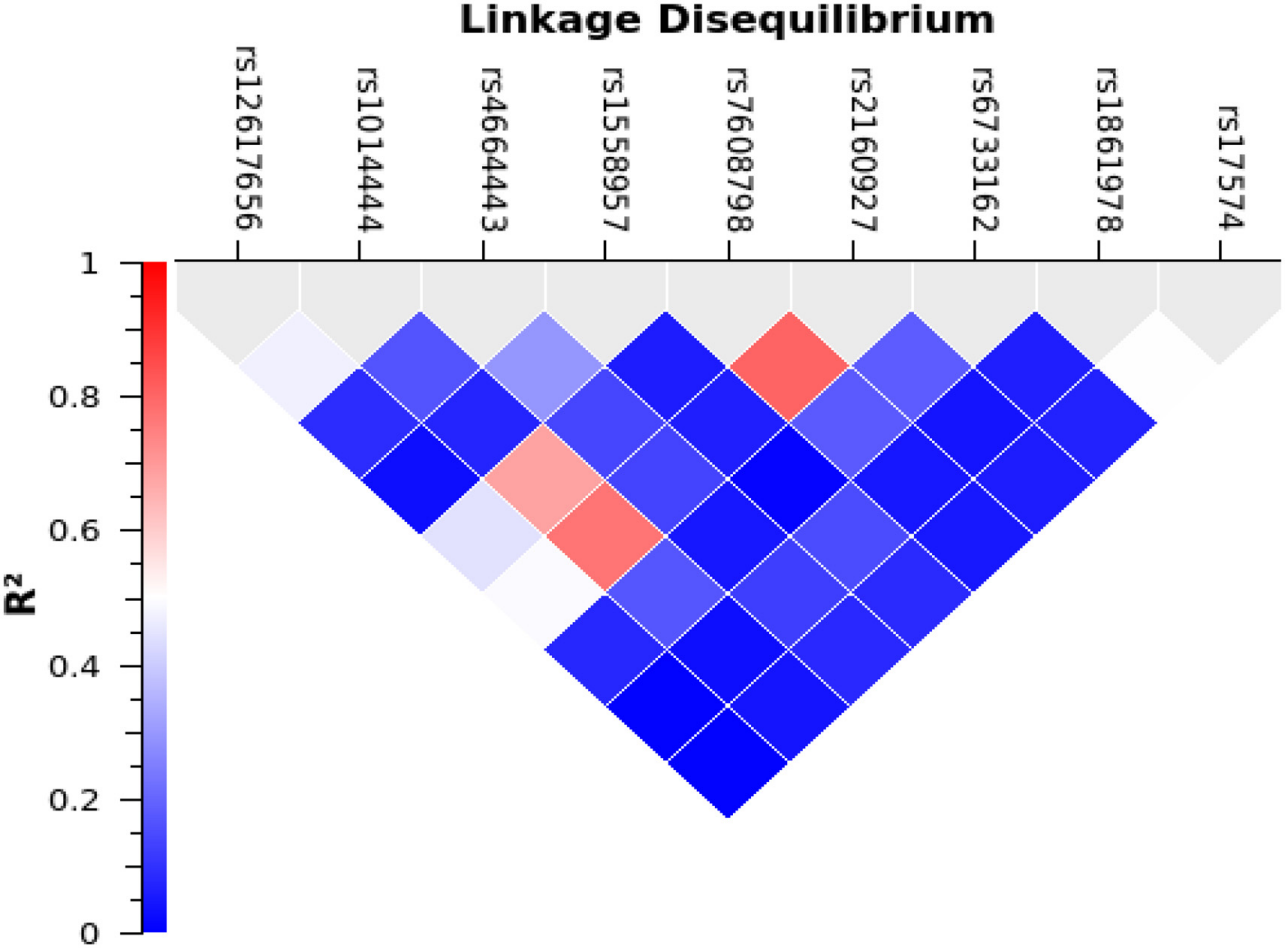
Pairwise linkage disequilibrium among DPP4 SNPs in Malaysian subjects. Values in the upper represent DPP4 SNPs, while values in the left represent the R^2^ value.

The linkage disequilibrium (LD) between SNPs were performed with SNP & Variation Suite (SVS) software. The degrees of LD with r^2^ among DPP4 SNPs are shown in Fig 1 and Table 3. It is noteworthy that a linkage disequilibrium block was not observed among these SNPs. Notably, these SNPs of DPP4 are an independent effect associated with T2DM in the Malaysian subjects (Table 3).

**Table 3:** The estimated values of linkage equilibrium analysis between the DPP4 SNPs in Malaysian Subjects.

D'	rs12617656 (C < T)	rs1014444 (G < A)	rs4664443 (G > A)	rs1558957 (C > T)	rs7608798 (A < G)	rs2160927 (T < C)	rs6733162 (G < C)	rs1861978 (G < T)	rs17574 (T < C)
rs12617656 (C < T)	-	0.71	0.71	0.59	0.69	0.72	0.29	0.27	0.21
rs1014444 (G < A)	0.47	-	0.94	0.86	0.88	0.88	0.44	0.54	0.53
rs4664443 (G > A)	0.09	0.17	-	0.76	0.91	0.90	0.53	0.48	0.32
rs1558957 (C > T)	0.03	0.07	0.30	-	0.81	0.85	0.36	0.40	0.36
rs7608798 (A < G)	0.44	0.68	0.14	0.06	-	0.90	0.42	0.68	0.65
rs2160927 (T < C)	0.49	0.78	0.13	0.06	0.80	-	0.43	0.68	0.65
rs6733162 (G < C)	0.08	0.17	0.05	0.01	0.18	0.18	-	0.84	0.72
rs1861978 (G < T)	0.01	0.03	0.12	0.15	0.04	0.04	0.06	-	0.85
rs17574 (T < C)	0.01	0.04	0.08	0.08	0.06	0.06	0.07	0.50	r^2^

The correlation cofficients D'and r^2^ between SNPs are shown in the above and below diagonal of this table, respectively.

### Association of DPP4 polymorphisms with T2DM among ethnic groups

The association of DPP4 SNPs with T2DM subjects among the three main Malaysian races (Malay, Chinese and Indian), which was assessed using logistic regression (adjusted for age, gender and BMI) revealed that rs12617656 was associated with T2DM among Malaysians of Indian ethnicity (recessive, OR (95%CI) = 3.21 (1.21–8.62); *p* = 0.019), dominant model OR (95%CI) = 3.72 (1.23–10.43); *p* = 0.003) and additive OR (95%CI) = 2.29 (1.24–4.24) *p* = 0.0009). Although rs7633162 showed a significant association among Malaysians of Malay ethnicity (additive, OR (95%CI) = 1.53 (1.01–2.30); *p* = 0.044) with T2DM, this association did not remain significant after controlling for a false discovery rate of 5%. (Table 4).

**Table 4:** Association of DPP4 polymorphisms with type 2 diabetes mellitus among Malaysian ethnic groups.

	Control		Type 2 diabetes		Recessive			Dominant			Additive		
DPP4 SNPs	Freq.	11/12/22	Freq.	11/12/22	OR (95% CI)	P-Value	P-Value*	OR (95% CI)	P-Value	P-Value*	OR (95% CI)	P-Value	P-Value*
rs12617656 (C < T)													
Malay^#^	0.49	24/40/22	0.56	27/82/44	1.50 (0.74–3.03)	0.258	0.028	1.99 (0.99–4.21)	0.051	0.009	1.52 (0.98–2.36)	0.059	0.011
Chinese^#^	0.38	20/25/7	0.49	16/22/15	2.16 (0.65–7.13)	0.208	0.022	1.05 (0.40–2.73)	0.924	0.048	1.30 (0.67–2.50)	0.439	0.041
Indian^#^	0.50	10/6/10	0.79	9/29/70	3.21 (1.21–8.62)	**0.019**	0.006	3.72 (1.23–10.43)	**0.003**	**0.004**	2.29 (1.24–4.24)	**0.0009**	**0.002**
rs7633162 (G < C)													
Malay^#^	0.53	24/33/29	0.58	31/67/55	1.67 (0.87–3.22)	0.123	0.015	1.97 (0.97–4.02)	0.062	0.013	1.53 (1.01–2.30)	**0.044**	0.007
Chinese^#^	0.44	17 /25/10	0.58	15/23/15	2.00 (0.71–5.61)	0.190	0.020	1.78 (0.66–4.80)	0.256	0.024	1.60 (0.86–2.99)	0.139	0.019
Indian^#^	0.77	1/11/14	0.72	9/42/57	0.99 (0.39–2.50)	0.979	0.050	0.37 (0.04–3.24)	0.366	0.035	0.86 (0.41–1.178)	0.675	0.046
rs4664443 (G > A)													
Malay^#^	0.13	66/19/1	0.14	117/30/6	3.35 (0.55–52.1)	0.123	0.017	1.30 (0.63–2.68)	0.472	0.043	1.43 (0.77–2.66)	0.256	0.026
Chinese^#^	0.06	48/3/1	0.08	47/5/1	3.32 (0.18–60.3)	0.418	0.037	1.83 (0.40–8.43)	0.436	0.039	1.71 (0.54–5.34)	0.360	0.033
Indian^#^	0.35	11/12/3	0.43	35/61/12	1.41 (0.32–6.1)	0.649	0.044	1.61 (0.62–4.17)	0.328	0.030	1.41 (0.69–2.91)	0.346	0.031

Risk allele frequency (Freq.) and genotype counts in individuals with type 2 diabetes and control subjects. In the additive model, genotype of homozygote for the non-risk allele (11), heterozygote (12) and homozygote for the risk allele 22. The recessive model was defined as 22 versus 12 + 11 and dominant model as 22+12 versus 11. The results are presented as odds ratio, 95% CI, and P value. adjusted for age, gender and BMI using logistic regression. The outliers (studentized residual is greater than 2.0 or less than −2.0) were excluded.

* Adjusted for multiple comparisons using the false discovery rate at the 5% level. Bold values are significant.

### Association of genotype groups in DPP4 (SNPs) with sDPP-IV levels

The association of genotype groups in DPP4 (SNPs) with sDPP4 levels were performed using the general linear model with a Bonferroni correction after adjusting for age, race, gender and BMI. Univariate analysis showed that the SNP rs4664443 G>A polymorphism in subjects with T2DM was associated with increased sDPP-IV levels (*p* = 0.042). Moreover, pairwise comparison, showed that the subjects with T2DM carrying the homozygous AA genotype of rs4664443 showed significantly higher serum sDPP-IV levels than subjects carrying the GG genotype (1346± 167 vs. GG 1215± 246 ng/mL, *p* = 0.039) (Table 5).

**Table 5:** Association of rs4664443 polymorphism with sDPP-IV levels among with type 2 diabetes mellitus subjects.

Parameter	Mean +SD			Univariate P-Value	Pairwise comparisons P-Value	
	A–A n(19)	A–G n(96)	G–G n(199) Ref		A–A *vs* G–G	A–G *vs* G–G
**sDPP**-**IV (ng/ml)**	1346 ± 167	1220 ± 232	1215 ± 246	**0.042**	**0.039**	**0.999**

The results presented represent as means ± SD, adjusted for age, gender, race, and BMI as covariates which were evaluated using univariate (General Linear Model) with a Bonferroni adjustment applied for multiple comparison tests. Ref: reference, the protective genotype was selected to be a reference for the comparison. Bold values are significant.

## Discussion

Over the past decade with the development of the genome-wide association studies (GWAS), a dramatic increase in T2DM susceptibility loci (from 5 in 2007 to 100) was reported, especially among European populations [27–32]. In a previous work on Malaysian subjects with T2DM, it was found that the serum sDPP-IV level increased in T2DM patients and correlated negatively with the levels of active GLP-1 [18]. However, the association between DPP4 polymorphism and T2DM has rarely been investigated in general [14], and no studies in the field have been carried out in Asian populations. Accordingly, there was a need to examine SNPs among Malaysian subjects with T2DM. The Malaysian population largely reflects the genetic diversity across Asia (Malay, Chinese and Indian). The current study is a novel contribution to investigate whether DPP4 polymorphism in Malaysian subjects is associated with T2DM, and to evaluate the SNPs within the DPP4 gene which, could be associated with the sDPP-IV levels.

Our results showed that certain DPP4 polymorphisms are associated with T2DM in the Malaysian subjects. The association was found at SNPs; rs12617656, rs7633162 and rs4664443. Such an association was more evident among the Indian ethnic group with respect to rs12617656. On the other hand, the SNP, rs4664443 was associated with sDPP-IV levels.

It is well-known that the inflammation was widely identified as a major contributor in the development of insulin resistance (IR) and T2DM [33–35], through activation of T-cells that mediate insulin resistance and adipose tissue inflammation [36], in which DPP4 plays a major role [37, 38]. DPP4 is upregulated in proinflammatory states including T2DM and obesity [17]. The T-cells activation involves the binding of DPP4 on the surface of T-cells to several matrix proteins including adenosine deaminase (ADA) and coassociation with CD45 resulting in costimulatory signals [17, 39, 40]. In addition, the secretion of DPP4 from skeletal muscle cells and adipocytes may exert paracrine effects on insulin signaling [41]. A new GWAS study by Jiang et al. [42] indicated that SNP rs12617656 at the DPP4 locus was associated with Rheumatoid Arthritis (RA) in the Han Chinese population, which might indicate the possible association between the inflammatory responses and rs12617656 in RA. In line with this, our study found a strong association between rs12617656 at the DPP4 locus and T2DM. Hence, it is noteworthy that chronic systemic inflammation has a key role in the pathophysiology of T2DM and RA [43], which can be explained by the elevated levels of inflammatory mediators such as C-reactive protein (CRP) and interleukin (IL)-6 in patients with RA [44], similar to T2DM [45].

In addition, we observed that SNP rs12617656 showed a stronger association with T2DM in Indians. Such an association might be attributed the gene-environment and gene-gene interactions that might have contributed to the differences in gene-disease associations among the different ethnic groups [46].

We also observed that SNP rs4664443 was associated with T2DM and linked to variations in the sDPP-IV levels. Dyslipidemia is a common feature of T2DM, and quantifiable by the measurement of apolipoprotein B (ApoB) levels [47]. A recent study by Baileys et al. [47] identified novel SNPs in DPP4 gene associated with ApoB levels in South Asian populations, who are prone to develop T2DM and MI at lower BMI and younger ages. They reported that SNP rs4664443 of DPP4 was significantly associated with ApoB levels. DPP4 and ApoB implicating with inflammation and inflammatory markers will help us understand how genetic variation in DPP4 gene contributes to susceptibility to T2DM. With respect to ApoB, it is found to be a predictor of inflammation and considered to be more closely related to inflammatory markers than the total, non- high-density lipoprotein (HDL), and low-density lipoprotein (LDL) cholesterol [48]. The research by Faraj et al. reported that higher levels of ApoB were associated with an increase in the levels of diverse inflammatory markers such as IL-6, tumor necrosis factor alpha (TNF-α) and CRP [49]. Furthermore, Ley et al. demonstrated that elevated ApoB concentration was associated with incident T2DM and was superior to HDL and LDL cholesterol in predicting the disease [50], and an effective predictor for the development of MetS [51]. Another study reported that insulin resistance was associated with the serum ApoB levels [52].

This study also demonstrated that SNP rs7633162 was significantly associated with T2DM. Alencia Vanay Woodard-Grice et al. analyzed the impact of DPP-IV inhibition on genotypic variation in DPP4, and showed that the haplotype of SNPs, rs673316-rs212469968-rs873826, was associated with a sitagliptin-induced decrease in DPP4 activity [53].

Finally, our results demonstrated that SNPs, rs1558957, rs7608798, rs17574 were not associated with T2DM. These findings were consistent with the study of Bouchard et al. [21], who conducted a multi-stage analysis for the association between DPP4 genetic polymorphism and cardiovascular disease risk factors, such as lipids, diabetes-related phenotypes, and blood pressure in European ancestry.Thus showing that rs17848915 now merged into rs17574, rs1558957 and rs7608798 of the analyzed SNPs were associated with hyperglycaemia/diabetes, triacylglycerol and LDL-cholesterol in the first stage, but were not successfully replicated in stage 2.

The limitations of this study are that it is a hospital-based study with a relatively small sample size, and the sampling method was non-probability. The sub-grouping of subjects according to races resulted in small sample size groups, which likely provides insufficient power to provide evidence for association within the ethnic subgroups. Thus, further studies may be warranted with a bigger sample size to better establish the association of DPP4 polymorphism with T2DM.

## Conclusion

Our results indicated that DPP4 genetic polymorphisms were associated with T2DM in the Malaysian subjects and linked to variations in sDPP-IV levels. In addition, these associations were more pronounced among Malaysian Indian subjects. Further studies with functional investigations will be necessary to confirm the association between the DPP4 genetic polymorphisms and T2DM.
